# Development and preliminary evaluation of a novel physician-report tool for assessing barriers to providing care to autistic patients

**DOI:** 10.1186/s12913-021-06842-1

**Published:** 2021-08-26

**Authors:** Chloe Walsh, Sinéad Lydon, Rosemary Geoghegan, Cornelia Carey, Michael Creed, Lauren O’Loughlin, Ellen Walsh, Dara Byrne, Paul O’Connor

**Affiliations:** 1 Department of General Practice, School of Medicine, National University of Ireland, Galway, Ireland; 2 Irish Centre for Applied Patient Safety and Simulation, University Hospital Galway, Galway, Ireland; 3 School of Medicine, National University of Ireland Galway, Galway, Ireland; 4 Discipline of Paediatrics, School of Medicine, National University of Ireland Galway, Galway, Ireland; 5 Department of Liaison Psychiatry, Beaumont Hospital, Dublin 9, Ireland; 6 Galway University Hospital, Galway, Ireland

**Keywords:** Autism, Physicians, Healthcare access, Health equity, Reasonable adjustments, Health

## Abstract

**Background:**

Individuals on the autism spectrum face significant disparities in health and physicians often report difficulties in providing care to autistic patients. In order to improve the quality of care autistic individuals receive, it is important to identify the barriers that physicians experience in providing care so that these may be addressed. This paper reports the initial development and preliminary evaluation of a physician-report ‘Barriers to Providing Healthcare’ measurement tool.

**Method:**

An established taxonomy of healthcare barriers for autistic individuals informed the initial draft of a 22-item measurement tool. This measurement tool was distributed to physicians working in various healthcare specialties and settings. Exploratory factor analysis (EFA) was conducted to determine the construct validity of the tool; discriminant validity between, and internal consistency of, the resultant factors were assessed. Multiple regressions were used to explore variables potentially associated with barriers endorsed by physicians.

**Results:**

A total of 203 physicians were included in the analyses. The EFA resulted in a 17-item tool with three distinct factors which explained 37.6% of the variance: 1) Patient-related barriers (Cronbach’s α = 0.83; e.g., the patient’s reactivity to the healthcare environment); 2) Healthcare provider (HCP)/family-related barriers (Cronbach’s α = 0.81; e.g., a lack of providers willing to work with autistic patients); and 3) System-related barriers (Cronbach’s α = 0.84; e.g., there is a lack of support for patients and families). Discriminant validity between the factors was adequate (*r* < .8). The barriers that were most frequently endorsed as occurring ‘often’ or ‘very often’ included a lack of support for patients and families (endorsed by 79.9% of physicians); communication difficulties (73.4%); and a lack of coordination between services (69.9%). The regression analyses identified no significant associated variables.

**Conclusion:**

A preliminary version of a novel physician-report tool to assess barriers to providing care to autistic patients has been developed although further validation work is required. The use of this tool will help physicians to identify issues specific to different medical specialities and healthcare settings. This information may help identify the supports physicians require to recognise and implement the required accommodations. Future research which elucidates barriers to healthcare provision for autistic patients is required to support systemic change in healthcare so as to improve care experiences and health outcomes for people on the autism spectrum.

**Supplementary Information:**

The online version contains supplementary material available at 10.1186/s12913-021-06842-1.

## Background

Substantial inequities in health outcomes and healthcare access exist for people with disabilities [1, 2]. These inequities are not attributable to an individual’s disability, however, but rather reflect a disparity in the accessibility of healthcare, whereby people with disabilities have more difficulty accessing the healthcare they need than others [3]. Autism Spectrum Disorder (hereafter ‘autism’) is one specific condition in which significant health disparities exist [4, 5].

Autism is a neurodevelopmental condition characterised by persistent difficulties or differences in social interactions and communication alongside repetitive and/or restrictive patterns of behaviour [6]. Currently, prevalence is estimated at one in 54 children, and one in 45 adults in the USA [7, 8]. Global prevalence is currently estimated at approximately 1 in 160 persons and is rising [9]. Autistic^1^ individuals experience a high prevalence of medical and psychiatric co-occurring conditions, which can make care complex [11]. This may be one reason that autistic individuals present to both emergency and non-emergency healthcare services more often [12], are admitted to hospital more often [13], and have longer hospital stays than their neurotypical peers [14]. Yet, despite a higher rate of healthcare utilisation, autistic individuals tend to experience more unmet healthcare needs [15, 16], poorer health outcomes [11, 17], poorer healthcare related quality of life [18, 19] and higher mortality rates than others [20, 21]. These data suggest that a disparity in access to quality healthcare exists for autistic individuals [22].

A number of recent systematic reviews have identified a variety of complex barriers which exist for autistic individuals when accessing and receiving healthcare which are likely contributing to this disparity [23–25]. These barriers may be understood to exist across the healthcare system: at the level of the patient (e.g., barriers associated with autism-related characteristics such as communication difficulties), healthcare provider (e.g., insufficient autism knowledge), and the system/organisation (e.g., a lack of resources) [23].

Considering the high prevalence of co-occurring conditions, the poorer healthcare experiences of autistic individuals, and the multitude of barriers which are known to exist, there is a recognised and pressing need to improve healthcare services for this population [15, 26]. Indeed, autistic self-advocates have highlighted improving health services and physical health outcomes as a research priority [27].

Ensuring accessible and equitable healthcare for autistic individuals, as is legally mandated in the UK [28, 29], requires that physicians are equipped with the tools, knowledge, and resources they need to care appropriately for this patient population [30]. In order to ensure that this is the case, the specific difficulties physicians face when caring for their autistic patients first need to be understood. Although measures exist to facilitate exploration of healthcare-associated barriers among autistic adults [26], or the caregivers of autistic individuals [31], there is a lack of established methods for engaging staff about their experiences and perceptions. A number of previous studies have used HCP-reported measurement tools to assess barriers to providing healthcare to autistic individuals, however, they are typically limited in the array of barriers that they assess (e.g., [32–34]). HCP-reported tools are important because they can provide information which may be missed by relying on the patient or caregiver perspective alone. Further, many interventions developed to reduce barriers to healthcare for autistic individuals will likely take place in healthcare settings and so, the HCP perspective is needed to ensure that such interventions are feasible from a physician perspective and are targeting issues that are important to both physicians and autistic individuals.

Accordingly, the aims of this study were to: 1) develop a physician-report tool to assess a more comprehensive array of barriers which may occur across the healthcare system and report on some preliminary assessments of validity and reliability (Phase one of the current study); 2) to examine the barriers endorsed by physicians, and identify potential factors associated with these barriers (Phase two of the study).

## Methods

### Context

In Ireland, although there are a very small number of private sector mental health services for autistic individuals, there are currently no speciality clinics within physical healthcare for autistic individuals. Instead, autistic individuals attend physical healthcare services, and are cared for, in the same manner as the rest of the population.

### Design

This study used a cross-sectional survey design.

### Participants

Eligible participants were physicians working in primary, secondary, psychiatric, or tertiary care services across the Republic of Ireland, who had some experience of caring for autistic individuals. Guidance on the required sample size to conduct an EFA varies [35–37]. A common rule of thumb denotes a 10:1 ratio of participants to items, though in general, larger sample sizes are recommended to produce stable factor structure [38]. Therefore, the authors aimed to satisfy this rule of thumb at the minimum but to recruit as large a sample as possible to ensure sufficient power.

### Recruitment

A variety of non-probability sampling methods and recruitment strategies were employed in an effort to recruit as wide a sample as possible. Convenience sampling and voluntary response sampling included: 1) circulating emails among staff within one medical school and one hospital group comprising of six hospitals; 2) placing advertisements in local and national newspapers, on local radio, and on social media; and 3) snowballing methods whereby participants were asked to share information about the study with others in their organisation(s) and/or social network. Anyone interested in the study contacted the researcher to request a survey pack or a link to an electronic version of the questionnaire.

### Data collection

Participants who completed paper forms were provided with pre-paid stamped return envelopes to return their surveys to the researchers. As an incentive, participants were offered the opportunity to enter a prize draw to win one of four gift vouchers to the value of €50 each.

### Ethical considerations

Ethical approval for this study was granted by the NUIG Research Ethics Committee (ref: REC 18-Jun-17). Informed written consent was obtained for each participant. For online surveys, participants clicked ‘I agree’ on an online consent form before being brought to the survey page.

### Phase 1. Tool development and evaluation

#### Literature review

A systematic literature review of 31 studies, was conducted to identify barriers to healthcare for autistic individuals and to develop a taxonomy of those barriers [23]. Included studies used quantitative, qualitative, and mixed methods, and participants included autistic individuals, caregivers, and HCPs. For quantitative studies, all questionnaire items which had been endorsed as barriers were extracted, while in qualitative studies, direct quotes and author-reported themes which described barriers were extracted. A thematic analysis approach was undertaken, initially guided by the domains outlined in Raymaker et al.’s [26] Barriers to Healthcare Tool. These domains included barriers related to: emotions, executive function, healthcare navigation, provider attitudes, patient-provider communication, sensory sensitivities, socio-economic issues, support, and waiting. A total of 320 individual barriers were identified across the studies. Not all barriers could be organised within the Raymaker et al. [26] domains and so new themes and subthemes were developed and organised into a taxonomy which consisted of three over-arching themes: 1) Patient-related barriers (autism-related characteristics; other patient-related barriers); 2) HCP-related barriers; 3) system-related barriers). Each theme had between two and eight subthemes (Table 1). Full details of this process are described elsewhere [23]. These themes and subthemes informed the item construction for the current tool.

**Table 1:** Themes and subthemes within the taxonomy which guided item development

Theme	Subtheme
Patient-related barriers: Barriers associated with autism-related characteristics	Communication/social difficulties
	Issues with waiting
	Issues with executive function
	Sensory issues
	Anxiety/other emotions
	Need for consistency
	Behavioural issues
Other patient-related barriers	Complexity of family involvement
	Scepticism towards conventional medicine
HCP-related barriers	Lack of autism knowledge/skill
	HCP inflexibility
	Stigma/negative perceptions
	Difficulties interpreting behaviour/symptoms
	Ignoring patient/caregiver concerns/expertise
	Poor HCP communication/failure to adapt language
System-related barriers	Lack of support for patients/caregivers
	Lack of support for HCPs
	Time/resource constraints
	Lack of continuity/collaboration between HCPs/services
	Location issues
	Financial/insurance issues
	Lack of qualified personnel
	Inflexible HC system

#### Item construction

Best practice in questionnaire design guided the development of the items for instrument development [39]. An iterative method was used to construct the items of the questionnaire. This involved two consensus building meetings between three members of the research team (CW, SL, POC). Specific items from existing questionnaires focused on barriers to healthcare for autistic patients were deliberately not reviewed as part of the item construction process as the team focused on the themes and subthemes of the taxonomy which had resulted from the prior systematic review [23]. In the first meeting, questionnaire items (*n* = 45) were constructed. The items were then reviewed, refined, and condensed into 22 items, each representing a different barrier linked to a subtheme. This process involved the research team discussing the items in detail and working together to identify items which were measuring the same subtheme. During a subsequent meeting, this process of reviewing the items was repeated to ensure all previous decisions were confirmed and to make any final refinements. This initial reduction in the number of items was conducted in order to avoid repetition among items and to reduce respondent burden. This is in line with best practice which recommends that measurement tools should be ‘usable’ (i.e. short, readable, and easy to complete) in order to avoid the potential for errors or non-response [40]. Efforts were made to reflect barriers which might occur at the level of the patient, HCP, and system*.*

#### Frequency and severity of barriers

For each item within this tool, respondents were asked to indicate the frequency with which each barrier had occurred in the past 12 months, rated on a Likert scale of 0 (never) to 4 (very often). Although it is generally recommended [41], a more concrete frequency scale (e.g., hourly, weekly, monthly etc.) was not utilised as it was expected that participants would have varying levels of contact with autistic patients. This made it difficult to frame frequency of contact around specific time periods. The options of ‘hourly or weekly’ for example, would not be applicable to a physician who only sees autistic patients occasionally. However, if a particular barrier was encountered every time the physician met an autistic patient, even if they only occasionally meet autistic patients, this might be considered a frequent barrier. Therefore, it was not deemed appropriate to use objective time intervals as these would only work if all participants were seeing autistic patients with roughly the same frequency. A recall period of 12 months was chosen as, although a shorter recall period (e.g., 6 months) may improve recall performance in some cases, such short recall periods may not capture infrequent events or behaviours [42–44]. As the respondents were anticipated to have fairly infrequent interactions with autistic patients, a 12 month recall period was chosen as the most appropriate recall period for this study.

The level of perceived severity (i.e., how much of a problem the barrier presented for the respondent) each barrier posed was rated on a Likert scale of 1 (slight), 2 (moderate), or 3 (severe). The response options were adapted from the Behaviour Problems Inventory [45] and the way in which the responses were presented in the tool was modelled on the Behaviour Problems Inventory. This presentation was chosen as it allowed assessment of both frequency and severity in tandem, rather than repeating the survey items twice (i.e., once for frequency and again for severity). It was hoped this would place the least burden on the respondent.

#### Statistical analyses

All statistical analyses were conducted in IBM SPSS (version 21). Significance levels for all analyses were set as *p* < .05.

##### Factor analysis

Factor analysis refers to a set of statistical procedures that can be used to identify the underlying constructs or domains that exist in a tool that is being developed [39, 43]. Factor analysis can take two forms: Exploratory Factor Analysis (EFA) and Confirmatory Factor Analysis (CFA). An EFA was chosen for this analysis rather than a CFA for a variety of reasons. First, it is both common and recommended that, on initial development of a new tool, an EFA should be conducted as a first step in assessing construct validity of a measure, even when existing literature and a priori hypotheses regarding factor structure to guide tool development exists [39, 46–48]. Second, EFA is used to identify latent constructs when there is insufficient evidence to make strong assumptions about the relationships among the items, how many common factors exist or what specific variables these common factors are likely to influence [46, 49, 50], as was the case in the current study. CFA, on the other hand, is conducted when a substantial theoretical base already exists, or when the relationship between items has already been tested and the factors and related items are known [51, 52]. CFA is typically used after an EFA, with a new data set, to assess the goodness of fit of a model when there is a strong model assumption [53]. Third, our aim was not to test the taxonomy, but to continue to refine the theory surrounding barriers to care. Thus, EFA was considered the more appropriate analysis to undertake in the current study due to the exploratory nature of the study, a lack of a sufficiently strong theoretical assumption of the model structure and the relationship between the items, and our aim of refining theory rather testing it [46, 49].

##### Initial data screening

Little’s test for Missing Completely at Random, and Missing Data Analysis as applied in IBM SPSS was used to assess the missing data.

##### Construct validity

Exploratory factor analysis (EFA), a reduction technique that enables the determination of the common latent variables that underlie the various items in a scale [54] was used to determine the construct validity of the Barriers to Providing Healthcare tool developed by the researchers. The EFA was conducted in accordance with best practice [38], and proceeded through the following steps:

Step 1: Adequacy of the correlation matrix.

Suitability of the data to an EFA was assessed by considering the sample size, factorability of the constructs (correlation matrix), examination of the Kaiser-Meyer-Olkin (KMO) Measure of Sampling Adequacy (MSA) and Bartlett’s Test for Sphericity.

Step 2. Factor extraction, retention, and interpretation.

Principal Axis Factoring was chosen as the factor extraction method as this method has an explicit focus on latent factors, whereas principal component factors, another common method, is computed without regard to any underlying structure caused by latent variables [38]. Factor extraction was determined by considering Kaiser’s criteria (Eigenvalue > 1), the scree plot, and a parallel analysis (PA) which was conducted via an online PA engine [55]. Oblique (Promax) rotation was used as the data cannot be assumed to be completely independent of each other and this is considered most accurate for research involving human participants [38, 48]. Through an iterative process, items were removed if they loaded onto more than one factor with a value > 0.4 or had weak loading values of < 0.4 [56, 57]. The pattern matrix guided interpretation and naming of the factors by the research team [56, 58].

Step 3. Discriminant validity and internal consistency of the factors.

Discriminant validity between the generated factors was assessed by examining the factor correlation matrix, with values < 0.8 indicative of adequate discriminant validity*.* Internal consistency of each of the generated factors was assessed using Cronbach’s alpha, with values > 0.7 indicative of good internal consistency [59].

### Phase 2. Assessment of barriers

#### Measurement tool

The survey instrument administered to participants consisted of three sections: 1) perceived barriers to providing care to autistic patients (i.e., the novel tool developed in Phase One); 2) physician knowledge of autism; and 3) demographics.

##### Frequency/severity of barriers

The measurement tool described in Phase one was administered. The tool contained 17 items which corresponded to individual barriers. Participants were asked to rate the perceived frequency and severity of each barrier presented. Subscale scores were calculated by summing the items in each subscale with higher score indicating more problems with the barriers.

##### Knowledge of autism

To assess participating physicians’ knowledge of autism, a 22-item Knowledge of Autism Scale, which uses a ‘true/false’ response option, was used [60, 61]. This scale assesses the participant’s knowledge of early signs of autism, descriptive characteristics, and commonly co-occurring behaviours. To score the scale, eight items are reverse scored and then all items are summed to obtain a total scale score. A score of 1 is attributed to true answers and a score of 0 to false answers. This scale has previously demonstrated moderate internal consistency (Cronbach’s α = 0.54) [61]. The internal consistency of the Knowledge of Autism scale in the current study was assessed by calculating Cronbach’s alpha.

##### Demographics

Physicians were asked to provide information on their sex, years of clinical practice, medical specialty, prior training in relation to autism, and the approximate number of autistic patients they treat per annum (Table 2).

**Table 2:** Respondent characteristics

Respondent Characteristics	***N*** (%)
*Sex*	
Female	116 (57.1)
Male	83 (40.9)
Prefer not to say	2 (1.0)
Other	2 (1.0)
*Level of seniority*	
Intern	34 (16.7)
SHO	46 (22.7)
Registrar	53 (26.1)
GP Trainee	3 (1.5)
GP	34 (16.7)
Consultant	33 (16.3)
*Years since graduation*	
< 5	81 (39.9)
5–10	54 (26.6)
11–20	39 (19.2)
21–30	11 (5.4)
> 30	18 (8.9)
*Autism training received*	
Undergraduate education	95 (46.8)
Postgraduate education	53 (26.1)
Continued medical education	42 (20.7)
Other	12 (5.9)
Never	49 (24.1)
*No. of autistic patients annually*	
< 10	142 (70.0)
10–30	41 (20.2)
31–60	17 (8.4)
61–100	2 (1.0)
> 100	1 (0.5)
*Medical specialty of participants*	
General practice	37 (18.2)
Paediatrics	28 (13.8)
Psychiatry	27 (13.3)
General Internal Medicine	20 (9.9)
Surgery	10 (4.9)
Geriatrics	7 (3.5)
Neurology	6 (3.0)
Emergency Medicine	4 (2.0)
Anaesthesia	4 (2.0)

*Note:* Numbers under the autism training category do not add to 203 because participants could choose more than one option; Numbers in under medical specialty do not equal 203 because not all respondents provided this information and interns are not included in this category; Levels of seniority are listed in ascending order

*GP* General practitioner, *SHO* Senior house officer

#### Statistical analyses

All statistical analyses were conducted in IBM SPSS (version 21). Significance levels for all analyses were set as *p* < .05.

##### Missing data analysis

Littles Test for Missing Completely at Random was used to assess the missing data. Where data were missing, simple mean imputation was used to replace the missing values [62] which allowed for the production of subscale scores needed for analysis.

##### Factors associated with barriers endorsed

In order to assess variables potentially associated with barriers, three hierarchical multiple regressions were conducted. Preliminary analyses were performed to ensure no violations of the assumptions of normality, multicollinearity, and homoscedasticity. The regressions assessed whether the frequencies of barriers were associated with the following variables: 1) medical specialty; 2) years since graduation from medical school; 3) attendance at autism training; and 4) autism knowledge. The number of autistic patients seen per year was controlled for within the regressions as it might be expected that physicians who see a higher number of autistic patients may report a higher frequency of barriers. The same method was used to complete all three regressions with just the criterion variable (i.e., patient-related barriers subscale; HCP/family-related barriers subscale; system-related barriers subscale) changed in each case.

## Results

### Response rate

As a variety of recruitment methods were used (e.g., leaflets, social media), it is not possible to provide an entirely accurate response rate. However, a total of 400 paper surveys were distributed and 226 were returned– an estimated response rate of 55.6%.

### Participants

A total of 23 participants did not provide any data for the Barriers to Providing Healthcare section and so were removed from all analyses, leaving a final sample of 203 physicians. The characteristics of these participants are presented in Table 2.

### Phase 1. Tool development and evaluation

#### Initial data screening

Of the Frequency of Barriers scale, 8.4% of the data were missing. Little’s test of missing completely at random indicated however, that the data were missing at random (χ^2^ = 249.162, *df* = 250, *p* = .503). No items were highly skewed or kurtosed (i.e., <− 2/> 2) [59, 62]. Simple mean imputation was therefore used to replace the missing data [62]. Inspection of the Severity scale indicated that a large amount of data were missing per item (*M* = 18.45%; *SD* = 4.07%; range 14–30.5%) and this appeared to be an artefact of the way the scale had been presented. This was considered to compromise the data that resulted from this scale. Therefore, the EFA was run using the Frequencies of Barriers data only.

#### Construct validity

##### Assessment of the adequacy of the correlation matrix

On examination of the correlation matrix two pairs of items were highly correlated (> 0.7). As a result, two items were deleted, one from each pair. The retained items made more theoretical sense based on barriers more commonly reported in the extant literature (e.g., a lack of coordination between services was retained instead of a lack of access to autism specialists). Subsequent examination of the correlation matrix suggested multicollinearity was unlikely to be an issue [58]. Bartlett’s test of sphericity indicated the correlation matrix was not an identity matrix (χ^2^ = 213.836, *df* = 190, *p* < .001). The Kaiser-Meyer-Olkin (KMO) Measure of Sampling Adequacy also indicated that the matrix was suitable for EFA (KMO = 0.91). Examination of the MSAs along the principal diagonal of the anti-image correlation matrix indicated that all items were suitable for inclusion in the EFA as all had a value greater than 0.8 or 0.9, and all off-diagonal values were small (< 0.2) [35].

##### Factor extraction, retention, and interpretation

Based on the Eigenvalue > 1 rule, five factors were extracted, the Parallel Analysis extracted three factors and the scree plot extracted 2 factors. The Kaiser Eigenvalue > 1 rule is not recommended as it has a tendency to over extract factors [35]. Therefore, follow up analyses were run extracting: 1) two factors based on the Scree plot, and 2) three factors based on the Parallel Analysis. When situations arise in which the various procedures suggest different numbers of factors, or when the procedures produce somewhat ambiguous results, it is recommended that the researcher examine the subset of models produced to assess which solution produces the most readily interpretable and theoretically sensible pattern of results [46]. After careful consideration of the two produced models, the three factor model produced by the Parallel Analysis made more theoretical sense as the items that clustered together were better interpreted as patient-related, HCP/family-related, and system-related. The two factor model is presented in Additional file 1. Correlations between the factors were observed in the factor matrix suggesting that an oblique rotation (Promax) be maintained [58]. A number of iterations of this analysis were conducted to identify and remove redundant items and to ascertain the best model for the data. On the first iteration, one item loaded very similarly onto 2 factors and so was discarded from the analysis. On the second iteration, one item did not load onto any factor > 0.4 and so was discarded from the analysis. On the third iteration, one item cross loaded on to two factors with a difference of < 0.2 and so was discarded from analysis. On the next iteration all items had factor loadings > 0.4 with no cross loadings evident, so no further iterations were conducted. This final model explained 37.6% of the variance. Table 3 presents the three extracted factors with the corresponding items, factor loadings and the amount of variance explained by each factor. The items which were removed during the analysis are presented in Additional file 2.

**Table 3:** EFA of three factor solution based on parallel analysis

	Factor 1	Factor 2	Factor 3
**Factor 1. Cronbach’s alpha = 0.83 Variance explained: 37.6%**			
Challenging behaviours exhibited by patient	**.808**		−.186
The patient’s reactivity to the healthcare environment	**.797**	−.172	
There are communication difficulties	**.733**		
Lengthy waiting room times for patients on the autism spectrum	**.550**		
The patient’s use of outside providers	**.518**	.286	
Consultations are too short to accommodate patients on the autism spectrum	**.476**		.259
**Factor 2. Cronbach’s alpha = 0.81; Variance explained: 5.9%**			
There is a lack of clarity regarding GP remit/referral		**.717**	
There are financial disincentives due to the need for additional time with the patient	.299	**.658**	−.223
The patients’ family/caregivers are sceptical of conventional medicine (e.g., vaccines)		**.602**	
There is a lack of providers willing to work with patients on the autism spectrum		**.586**	.237
Family/caregiver involvement makes provision of healthcare to patients on the autism spectrum more complex		**.561**	.146
I prefer to avoid working with patients on the autism spectrum	−.158	**.554**	
**Factor 3. Cronbach’s alpha = 0.84; variance explained: 5.6%**			
There is a lack of support for families and patients			**.814**
The physical environment in healthcare settings is unsuitable	.170	−.102	**.719**
Lack of own knowledge for working with patients on the autism spectrum	−.191	.164	**.614**
There is a lack of coordination between services	.238		**.584**
There are shortages of medical and non-medical services for people on the autism spectrum	.272	.154	**.528**

*GP* General practitioner

##### Determination of discriminant validity and internal consistency of the generated factors

The factor correlation matrix indicated adequate discriminant validity between the two factors as the values were all < 0.8. As can be seen in Table 2, Cronbach’s alphas indicated that all factors showed good internal consistency (all > 0.8) as per conventional standards of interpretation [63].

### Phase 2. Assessment of barriers

#### Frequency scores

As can be seen in Table 4, the barriers most endorsed as occurring ‘often’ or ‘very often’ included: a lack of supports for patients and families (79.9%); communication difficulties (endorsed by 73.4% of respondents); and a lack of coordination between services (69.9% of respondents). More details on barriers endorsed are provided in Table 4.

**Table 4:** Number and percentage of respondents who endorsed each item

	Very often *N*(%)	Often *N*(%)	Sometimes *N*(%)	Rarely *N*(%)	Never *N*(%)
**Patient-related barriers**					
Challenging behaviours exhibited by the patient	17 (8.4)	94 (46.3)	62 (30.5)	23 (11.3)	7 (3.4)
The patient’s reactivity to the healthcare environment	28 (13.8)	108 (53.2)	42 (20.7)	17 (8.4)	8 (3.9)
There are communication difficulties	57 (28.1)	92 (45.3)	31 (15.3)	17 (8.4)	6 (3)
Lengthy waiting room times for patients on the autism spectrum	36 (17.7)	67 (33)	50 (24.6)	23 (11.3)	27 (13.3)
The patient’s use of outside providers	24 (11.8)	67 (33)	56 (27.6)	18 (8.9)	38 (18.7)
Consultations are too short to accommodate patients on the autism spectrum	38 (18.7)	68 (33.5)	53 (26.1)	21 (10.3)	23 (11.3)
**HCP/Family-related barriers**					
There is a lack of clarity regarding GP remit/referral.	26 (12.8)	55 (27.1)	50 (24.6)	22 (10.8)	50 (24.6)
There are financial disincentives due to the need for additional time with the patient	19 (9.4)	42 (20.7)	49 (24.1)	28 (13.8)	65 (32)
The patients’ family/caregivers are sceptical of conventional medicine (e.g., vaccines)	9 (4.4)	29 (14.3)	63 (31)	59 (29.1)	43 (21.2)
There is a lack of providers willing to work with patients on the autism spectrum	21 (10.3)	46 (22.7)	51 (25.1)	33 (16.3)	52 (25.6)
Family/caregiver involvement makes provision of healthcare to patients on the autism spectrum more complex	22 (10.8)	39 (19.2)	56 (27.6)	50 (24.6)	36 (17.7)
I prefer to avoid working with patients on the autism spectrum	12 (5.9)	25 (12.3)	48 (23.6)	59 (29.1)	59 (29.1)
**System-related barriers**					
There is a lack of support for patients/families	70 (34.5)	82 (40.4)	25 (12.3)	15 (7.4)	11 (5.4)
The physical environment in healthcare settings is unsuitable	53 (26.1)	87 (42.9)	38(18.7)	14 (6.9)	11 (5.4)
Lack of own knowledge for working with patients on the autism spectrum	34 (16.7)	96 (47.3)	40 (19.7)	23 (11.3)	10 (4.9)
There is a lack of coordination between services	65 (32)	77 (37.9)	29 (14.3)	19 (9.4)	13 (6.4)
There are shortages of medical and non-medical services for people on the autism spectrum	45 (22.2)	100 (49.3)	29 (14.3)	13 (6.4)	16 (7.9)

For the novel tool, subscale scores were calculated by summing the responses for each subscale produced by the EFA (i.e., Patient-related barriers; HCP/family-related barriers; system-related barriers). On average, physicians scored highest on the patient-related barriers subscale (*M* = 14.8, *SD* = 5.0; range 0–24). This was followed by the system-related barriers subscale (*M* = 13.8, *SD* = 4.3 range:0–20). HCPs scored lowest on the HCP/family related subscale (*M* = 10; *SD* = 5.5; range:0–24).

#### Severity scores

Due to a large amount of missing data on the severity scale, no analysis was conducted using these data.

#### Knowledge scores

Missing data analysis indicated that 10% of the data were missing from the knowledge scale. Little’s test of Missing Completely at Random indicated that the data were missing at random (χ^2^ = 207.519, *df* = 224, *p* = .778). Therefore, simple mean imputation was used to replace the missing values [62]. Internal consistency was deemed moderate (Cronbach’s *α* = 0.58). Physicians generally scored highly on autism knowledge (*M* = 18.5*, SD* = 2.2*,* range: 9–22).

#### Variables potentially associated with barriers

##### Assumptions of regression

Preliminary analyses indicated no violations of the assumptions of multicollinearity and homoscedasticity.

##### Variables associated with the frequency of patient-related barriers

The overall model, which included autism knowledge, medical specialty, years since graduation, and previous autism training, was not significant. Details of these findings are provided in Additional file 3.

##### Variables associated with the frequency of HCP/family-related barriers

The overall model, which included autism knowledge, medical specialty, years since graduation, and previous autism training, was also not significant (see Additional file 3).

##### Variables associated with the frequency of system-related barriers

The overall model, which included autism knowledge, medical specialty, years since graduation, and previous autism training, was also not significant (see Additional file 3).

## Discussion

In order to reduce inequities in access to healthcare, there is a need to make adaptions to health services and the delivery of care to accommodate autistic patients. In some countries, including the UK, this has become a legal requirement [28, 29]. However, physicians and healthcare organisations struggle to identify how best to adapt services or support autistic patients [30, 64, 65]. This paper reports the development of a novel physician-report Barriers to Providing Healthcare measurement tool that may be used to identify priority areas for change and which can support quality improvement activities. The data collected provide an important insight into physicians’ experiences of barriers to providing care to autistic patients.

### Phase 1. Tool development and evaluation

The EFA resulted in a 17-item tool for the assessment of barriers physicians experience when providing healthcare to their autistic patients. The tool consists of three subscales: patient-related factors (e.g., communication difficulties); HCP/family-related factors (e.g., HCP prefers to avoid working with autistic patients), and system-related factors (e.g., there is a lack of coordination between services) that demonstrate good internal consistency and construct validity. This tool is largely congruent with current understandings of barriers to healthcare access experienced by autistic patients and their caregivers [26, 66], and may be considered to supplement existing tools for caregivers [31] and autistic adults [26]. It must be noted that there is some convergence between items in the current tool and those of previously reported tools which assess barriers to healthcare for autistic patients (e.g., [13, 33, 61, 67]). This is to be expected since the items were based on a taxonomy that was informed by the existing literature and there is only a finite number of ways in which to describe a given barrier.

The current data align relatively well with the systematic review derived taxonomy used to guide the tool development. The systematic review found three distinct themes: 1) patient-related barriers; 2) HCP-related barriers; and 3) system-related barriers. Although the EFA in the current study also produced three factors, some discrepancies emerged in how some items organised within the factors resulting from the EFA. For example, lengthy waiting room times and consultations being too short loaded on to the patient-related factor when it might have been expected that they would load on to the HCP- or system-related factor. This suggests that physicians in the current sample perhaps interpret these factors as patient-related because, although they might exist for all patients, these barriers become a much more significant problem, and impact on care more greatly, when associated with autistic patients in comparison to non-autistic-patients. This added complexity specifically related to autistic patients has been commonly described by HCPs as a challenge in the literature [68]. Further, a lack of autism knowledge among HCPs loaded onto the system-related factor, whereas in the taxonomy, this is an HCP-related factor. It is possible that HCPs interpret this as a system-related factor as they potentially feel that they do not have access to adequate training [61]. A lack of clarity over GP remit/referral pathways and financial disincentives both loaded onto the HCP/family-related factor when they might have been expected to load onto the system-related factor. It is unclear why this occurred, but the findings support previous research that has indicated that clinicians are sometimes less likely to see the system-related causes of problems [69]. This suggests that more work is needed to help physicians identify and interpret system-related issues [69]. Finally, two items relating to the involvement of caregivers (i.e., family/caregiver involvement makes care complex; family/caregivers are sceptical of conventional medicine) both loaded with HCP-related items to create the HCP/family-related factor. It is possible that HCPs perceive caregivers as a type of care provider because caregivers tend to be so highly involved in the provision of, and decision-making regarding, the care of autistic individuals across the lifespan. These discrepancies indicate the need for further validation work on both the current tool and the taxonomy. For example, another EFA could be conducted with a larger sample to see if the current factor structure remains stable. A CFA could also be conducted to test the factor structure.

### Phase 2. Assessment of barriers

A recent taxonomy [23] elucidated how barriers to healthcare for individuals with autism may occur at various levels of the healthcare system. These findings are supported by the current study as physicians endorsed barriers at the level of the patient (e.g., communication issues), the HCP (e.g., HCPs unwilling to work with autistic patients), and the system (e.g., a lack of coordination between services). A lack of support for patients and families was most endorsed as occurring ‘often’ or ‘very often’ by HCPs in the current sample. This is consistent with existing literature. Warfield et al. [70], also reported that physicians in their study indicated that a lack of services and supports for autistic youth and adults was a challenge to providing care. Similar results were observed by Unigwe et al. [61] where physicians reported that their autistic patients were left unsupported due to a lack of joined up services which made referral difficult. In particular, GPs reported that there was a substantial lack of support for adults after receiving an autism diagnosis, and no supports for autism management or accessing therapy [61]. Some HCPs have suggested that practices could compile a list of local supports that could be given to their patients as one means of addressing this barrier [70]. However, adequate supports first need to be put in place. Thus, it is likely that, system-level changes are required to support autistic individuals and their families, and such changes must also consider how to improve referral pathways to any such supports [61].

Communication difficulties or differences are a diagnostic criterion for autism and can certainly become barriers in medical contexts [6]; for example, some individuals have difficulty expressing pain/symptoms [71] making interpretation difficult for the physician and/or caregiver [72, 73]. Further, some individuals may have slower language processing speeds, making it difficult to keep up with the typical pace of a conversation [26] and may result in the patient missing important medical information or not having ample time to ask questions [74]. Hospital passports, which allow the patient to communicate important information to the physician/healthcare facility (e.g., medications, allergies, sensory sensitives, or communication needs), have been shown to be helpful for facilitating communication between providers and patients with intellectual and developmental disabilities [75]. Although autistic self-advocates have endorsed hospital passports with the National Autistic Society UK [76] providing one version on their website, more empirical work is needed to examine the effectiveness of using hospital passports in improving communication between providers and their autistic patients.

A lack of coordination between healthcare services was the third most endorsed barrier. Due to the high prevalence of co-occurring conditions that many autistic individuals experience [11], their care can be complex and often requires the involvement of a variety of professionals, including mental health professionals, occupational therapists, physical therapists, speech and language therapists, social services, neurologists, and other clinical specialists [72]. Several studies have highlighted issues with care coordination in the context of autism. For example, parents of autistic children were three times more likely to identify issues with care coordination between specialty doctors and other providers than parents of children with other types of special healthcare needs [77]. Relatedly, physicians have noted difficulties such as identifying appropriate referral pathways as particularly problematic in relation to autistic patients [61, 72]. Addressing such barriers will require system-level changes in how information is shared across healthcare services and providers. Electronic health records and flagging systems have shown promising results in improving the flow of information [78] between providers and may warrant further investigation in relation to autistic patients [78–80]. The current tool could be used to assess in which contexts (e.g., primary care, neurology) care coordination is a more significant barrier and is therefore most in need of such interventions.

It was hoped that examining variables potentially associated with barriers (e.g., autism knowledge, years of clinical practice) would help to indicate where and for whom barriers are likely to occur so as to allow improvement efforts and resources to be most clearly directed. However, no clear associated variables emerged in our analysis. This echoes Nicolaidis and colleagues [64] who found no difference in self-efficacy scores among healthcare providers for caring for their autistic patients by medical specialty, academic degree, or autism training status. One potential reason for this is the possibility that factors such as level of clinical experience or specialty currently make little difference because any autism training received was too generalised or did not target the areas that physicians most require training in, such as how to identify and implement required accommodations. Unigwe and colleagues [61], for example, showed that although autism knowledge was high among their sample of GPs in the UK, perceptions of self-efficacy to manage the ongoing care of autistic patients was low. Further, Tuffrey-Wijne and colleagues [80] found that although HCPs were aware of the need for accommodations for patients with intellectual disabilities, they sometimes struggled to identify what those accommodations were. Therefore, training should incorporate information on common accommodations required within specific settings as a potential means to address this problem.

It is insufficient to focus on physician training alone, however. In the current study, a limited number of potential associated variables were examined, all relating to the HCP. Future research needs to consider system-level variables, such as the financial supports, that might predict responses. It is possible that system-level issues prohibit physicians from feeling empowered or supported to implement accommodations to overcome barriers. Tuffrey-Wijne and colleagues’ work highlighted organisational/system level barriers to adjusting care for people with intellectual disabilities that included a lack of clear lines of responsibility, and a lack of funding and resources for implementing accommodations [80]. Following further validation work, it is hoped that the current tool could be used to identify the specific systemic barriers that physicians across specialities and settings experience in relation to treating their autistic patients. It is hoped that this knowledge would guide how to best use the limited resources available in overcoming such barriers by highlighting the specific resources that would be most beneficial in different contexts; for example, funding for a multisensory room may be better allocated to a busy emergency department than a quiet out-patient service which can already provide a quiet area for an autistic individual to wait in until their appointment.

### Limitations

This study had a number of limitations which must be taken into consideration when interpreting the results. First, although the sample size was acceptable for an EFA, it was still relatively small. Tabachnick & Fidell recommend a sample size of ~ 300 in order to be ‘comfortable’ [35]. However, MacCallum and colleagues [81] suggest that sample sizes in the range of 100–200 are acceptable as long communalities are mostly in the 0.5 range and there are is high overdetermination of factors (e.g., at least six or seven indicators per factor and a small number of factors), as was the case in the current study. Regardless, a more stable factor solution may have been obtained with a larger sample size. Future research should investigate this.

A second limitation is the heterogeneity of the sample. It would be expected that physicians from different specialities would experience different barriers to different extents. However, with a lack of sufficient empirical research focused specifically on this to date [23], the tool was designed as a general measure for physicians of all specialities with the hope that this would allow comparisons to be made between specialities, to guide the development of more targeted tools in future. A further limitation related to the sample is that the majority of participants did not have regular contact with autistic patients, although similar rates of contact have been observed in existing literature [32, 33]. Due to the lack of specialist autism clinics within physical healthcare in Ireland, it was difficult to identify a specific group of physicians who would have high levels of contact with autistic patients. Therefore, efforts were made to recruit as large and varied sample as possible, in the hope that participants experienced in treating autistic patients would be identified. It is unclear why so many participants did not have much contact with autistic patients. It could be due to non-disclosure of the diagnosis in some cases [71, 74]. Low levels of contact do not necessarily mean low levels of knowledge, however, as the participants in the current sample scored highly on the Knowledge of Autism test. Further, all participants had some level of experience with autistic patients, and 60% of the current sample had more than 5 years’ clinical experience. Experience builds over time, so, although high levels of monthly contact with autistic patients was not observed, the participants still had valuable insight to offer. Nonetheless, future research administering the tool to a sample of physicians who are known to have a lot of experience with autistic patients is recommended.

A third limitation is that, although the tool was informed by previous qualitative and quantitative research involving autistic individuals, their supporters, and HCPs as synthesised within a systematic review [23], there was no direct involvement of the autistic community or physicians in the development of the tool. The authors recognise, however, that as research on the barriers to healthcare experienced by autistic individuals increases and tools are developed to represent the perspectives of patients, caregivers and HCPs, there is a need to engage in co-creation of tools and interventions with autistic individuals, HCPs and caregivers [82, 83]. Doing so will help to ensure that research priorities are aligned with the autistic community’s priorities [84]. Future validation work is recommended on the current tool which could include methods such as cognitive interviewing with autistic individuals and physicians to assess the validity of the items from their perspectives.

A fourth limitation is related to potential issues with the face validity of some items within the tool given the unexpected manner in which some items loaded. However, the discrepancies between how some items loaded and the organisation of the taxonomy have potentially highlighted an area of interest regarding how physicians perceive certain barriers, and this warrants further investigation.

A fifth limitation was that, although respondents were asked to report on the severity of the barriers endorsed (data not presented herein) as well as frequency, many respondents did not. It is likely that this was an artefact of how this scale was presented in the questionnaire (i.e., the frequency and severity scales were presented adjacent to one another; see Additional file 4). As there was so much missing data for this scale, this information was not analysed. Future research should conduct a CFA to examine the items of the current tool with the severity scale as severity is potentially a more important indicator than frequency in the assessment of barriers. A barrier that occurs frequently may not actually pose that much of an issue; however, a barrier that is perceived as severe, whether it occurs frequently or not, could have a much greater impact on the accessibility or experience of care and may, therefore, be more important to target within interventions. For example, lengthy waiting room times may occur very frequently, but do not pose significant issues for all autistic individuals. It would, therefore, be more helpful to know how severe this issue is for a particular individual before deciding whether to allocate resources to address this barrier for that individual. Examining the perceived frequency and severity of barriers in tandem could, therefore, offer a greater insight into the experiences of these barriers and thus, be helpful in guiding the prioritisation of attention and resources.

Finally, this paper describes the preliminary assessment of the tool, among an Irish cohort of physicians, only. Due to single administration of the tool, it was not possible to assess test-retest reliability. As there is a lack of fully validated similar physician-report tools for assessing barriers to providing care to autistic individuals, it was not possible to assess convergent/discriminant validity with other measures.

### Future research

It is hoped that this tool will eventually be used by physicians to identify the barriers they most commonly experience within their own contexts and use this to generate discussion about accommodations that might be required by their autistic patients. However, it is important to note that the current paper only describes the initial development and evaluation of the tool; further validation work is required. First of all, cognitive interviews should be conducted with physicians to assess the face and content validity of the items within the scale and to assess the presentation and interpretability of the response options. This should than be followed by further psychometric evaluations on the tool: A CFA should be conducted to assess the goodness of fit of the model [39], followed by further evaluations to examine: reproducibility (i.e., does the same factor structure result from the analysis of another sample of responses?); responsiveness (is the tool sensitive to changes?); interpretability (can qualitative meaning be assigned to the quantitative scores?) [39]; and usability (i.e., does the tool achieve the specified goals with effectiveness, efficiency, and satisfaction, in the specified context, for the specified end users [40]? Future research should also assess whether adaptations are required to suit different medical specialities (e.g., GPs vs surgeons), or healthcare settings (e.g., primary vs secondary care) and whether these might increase the ‘actionability’ of the resulting data. Future research could also assess whether the tool could be used as a means of benchmarking across specialties and settings and whether benchmarking considerations differ between different specialities. Finally, future work would also need to evaluate the tool in international populations in order to support its use in other countries and healthcare systems.

Future research should also use the current tool with other HCP populations such as nurses and allied health professionals. In a previous study [31], the authors developed a caregiver-report Barriers to Healthcare tool, and Raymaker and colleagues [26] have developed a self-report tool for autistic adults. There is a need however, to involve healthcare providers other than physicians in this type of work as these professionals are also highly involved in the care of autistic patients. Future work may want to use the current tool to assess whether the barriers they experience are different and whether tailored tools are required. Triangulation of this data with the data from the current study and other research with caregivers and autistic adults would provide a truly holistic view of the issues related to autism and healthcare.

In order to further improve the triangulation of data, other methods of assessing barriers are required. Although quantitative methods provide valuable insight, they are unlikely to produce a full understanding the underlying processes [85]. Qualitative approaches are, therefore, also recommended to gain a deeper understanding of how and why barriers manifest, as well as to identify potential solutions [86]. Patient narratives, which may be gathered through semi-structured interviews, are a recognised means of informing quality improvement initiatives in healthcare [86, 87] and so, should be explored by future research in order to complement and enhance the data gathered by quantitative measurement tools.

Finally, as tools to assess barriers to healthcare for autistic individuals are developed and implemented, there is a need to give consideration to the next step: how to actually implement changes to address the information collected. There are, therefore, two key considerations for future research. First, there is a need to identify and evaluate the evidence for interventions that have been trialled to date such as autism specific care plans [88] or online autism training for HCPs [89]. Second, there is a need for engagement with stakeholders (i.e., HCPs, autistic people, caregivers) to consider mapping of these interventions to the identified barriers and also to consider how to address barriers which are not clearly covered by existing interventions. There now exist a number of good measurement tools, therefore it is imperative that we move our focus to facilitating the use of the valid and reliable data collected by these tools to actually addressing the issues identified and improving the quality of care of autistic individuals.

## Conclusion

Best practice denotes that physicians and other HCPs provide accommodations to their autistic patients to ensure healthcare is accessible and equitable. The current paper has presented a preliminary version of a novel physician-report Barriers to Providing Healthcare tool which, after further evaluation and validation work, may be used in practice to help physicians distinguish the barriers that exist for them in specific healthcare contexts. Obtaining this information may help identify the supports physicians need to overcome these barriers, and to identify and implement the required accommodations. Finally, as information is gathered on barriers to healthcare for the autism community, there is a need, going forward, to translate this information into effective quality improvement initiatives regarding the care of autistic individuals.

## Supplementary Information

**Additional file 1.** Two factor solution with extracted factors, items, internal consistency, and variance explained

**Additional file 2.** Additional items that were removed during analysis

**Additional file 3.** Summary of regression analyses

**Additional file 4.** Final Barriers to Providing Healthcare measurement tool

## Data Availability

The dataset used and/or analysed during the current study are available from the first author upon reasonable request.

## Abbreviations

CFA: Confirmatory Factor Analysis
EFA: Exploratory Factor Analysis
GP: General Practitioner (family or primary care physician)
HCP: Healthcare Provider
KMO: Kaiser-Meyer-Olkin
MSA: Measures of Sampling Adequacy
PA: Parallel Analysis
SHO: Senior House Officer
UK: United Kingdom

## Footnotes

1 Use of identity-first language: many autistic individuals have indicated a preference for the use of identity-first language (i.e., autistic person) as opposed to person first language (i.e., person with autism [10]. Accordingly, this article will use identity first language.
