# Supplementary material for: Development and preliminary evaluation of a novel physician-report tool for assessing barriers to providing care to autistic patients
Source: BMC Health Serv Res. 2021 Aug 26;21:873. doi: 10.1186/s12913-021-06842-1 (PMC8390217; doi:10.1186/s12913-021-06842-1)
Supplement: Supplementary file 1 — Additional file 1. Two factor solution with extracted factors, items, internal consistency, and variance explained [file 12913_2021_6842_MOESM1_ESM.docx]

**Additional file 1.**

Two factor solution with extracted factors, items, internal consistency, and variance explained

| **Item** | **Factor 1** | **Factor 2** |
| --- | --- | --- |
| **Factor 1: Patient-related factors; Cronbach’s α=0.9; variance explained 38.9%** |  |  |
| The patient’s reactivity to the healthcare environment | **.830** | -.169 |
| There are insufficient recourses | **.752** | .038 |
| Consultations are too short to accommodate patients on the autism spectrum | **.744** | -.034 |
| There are communication difficulties | **.724** | -.044 |
| Lengthy waiting room times for patients on the autism spectrum | **.701** | -.048 |
| Challenging behaviours exhibited by the patient | **.655** | -.034 |
| Limited flexibility to accommodate patients on the autism spectrum and their needs | **.629** | .183 |
| There is a lack of coordination between services | **.569** | .157 |
| The patients’ use of outside providers (e.g., alternative therapies) | **.506** | .190 |
| The physical environment is unsuitable for patients on the autism spectrum | **.503** | .190 |
| **Factor 2: Provider and system-related factors; Cronbach’s α=0.8; Variance explained: 6.0%** |  |  |
| There is a lack of clarity regarding GP remit/referral | -.011 | **.742** |
| There is a lack of providers willing to work with patients on the autism spectrum | .008 | **.733** |
| There is a lack of guidelines for working with autistic patients | -.028 | **.716** |
| I prefer not to work with patients on the autism spectrum | -.170 | **.627** |
| The patient’s family is sceptical of conventional medicine (e.g., vaccines) | .074 | **.516** |
| Family/carer involvement makes provision of healthcare for patients on the autism spectrum more complex | .101 | **.513** |
| There are financial disincentives due to the need for additional time with the patient. | .196 | **.505** |
| Lack of own knowledge regarding autism. | .053 | **.478** |

GP=general practitioner;
