# Supplementary material for: Development and preliminary evaluation of a novel physician-report tool for assessing barriers to providing care to autistic patients
Source: BMC Health Serv Res. 2021 Aug 26;21:873. doi: 10.1186/s12913-021-06842-1 (PMC8390217; doi:10.1186/s12913-021-06842-1)
Supplement: Supplementary file 2 — Additional file 2. Additional items that were removed during analysis [file 12913_2021_6842_MOESM2_ESM.docx]

**Additional file 2.** Additional items that were removed during analysis – items are listed in the order in which they were removed.

|  | **Item** |
| --- | --- |
| 1 | There is a lack of access to autism specialists |
| 2 | Lack of confidence in own ability to care for people on the autism spectrum |
| 3 | There is a lack of guidelines for caring for people on the autism spectrum |
| 4 | There is limited flexibility to accommodate patients on the autism spectrum |
| 5 | There is a lack of resources |
