# Supplementary material for: Development and preliminary evaluation of a novel physician-report tool for assessing barriers to providing care to autistic patients
Source: BMC Health Serv Res. 2021 Aug 26;21:873. doi: 10.1186/s12913-021-06842-1 (PMC8390217; doi:10.1186/s12913-021-06842-1)
Supplement: Supplementary file 3 — Additional file 3. Summary of regression analyses [file 12913_2021_6842_MOESM3_ESM.docx]

**Additional file 3.** Summary of regression analyses

|  | **Variable** | ***F change*** | ***R*^2^** | ***A R*^2^** |
| --- | --- | --- | --- | --- |
| Variables associated with frequency of patient-related barriers | |  |  |  |
| 1. | No. of autistic patients seen per annum | 1.633 | .008 | .003 |
|  |  |  |  |  |
| 2. | Autism knowledge | .883 | .043 | -.002 |
|  | Medical specialty |  |  |  |
|  | Years since graduation |  |  |  |
|  | Previous autism training |  |  |  |
| Variables associated with frequency of HCP/family-related barriers | |  |  |  |
| 1. | No. of autistic patients seen per annum | .953 | .005 | .000 |
|  |  |  |  |  |
| 2. | Autism knowledge | 1.735 | .072 | .028 |
|  | Medical specialty |  |  |  |
|  | Years since graduation |  |  |  |
|  | Previous autism training |  |  |  |
| Variables associated with frequency of system level barriers | |  |  |  |
| 1. | No. of autistic patients seen per annum | .214 | .001 | -.004 |
| 2. | Autism knowledge | 1.626 | .064 | .021 |
|  | Medical specialty |  |  |  |
|  | Years since graduation |  |  |  |
|  | Previous autism training |  |  |  |

HCP=Healthcare provider
