# Supplementary material for: Development and preliminary evaluation of a novel physician-report tool for assessing barriers to providing care to autistic patients
Source: BMC Health Serv Res. 2021 Aug 26;21:873. doi: 10.1186/s12913-021-06842-1 (PMC8390217; doi:10.1186/s12913-021-06842-1)
Supplement: Supplementary file 4 — Additional file 4. Final Barriers to Providing Healthcare measurement tool [file 12913_2021_6842_MOESM4_ESM.docx]

**Additional file 4.** Final Barriers to Providing Healthcare measurement tool

|  | | **Frequency** | | | | | **Severity** | | |
| --- | --- | --- | --- | --- | --- | --- | --- | --- | --- |
| For each item, circle the number in the appropriate boxes to indicate:  **(A) how often** the described barrier typically occurs for you when providing healthcare to a patient on the autism spectrum; (*frequency*);  **AND**  **(B) how much of a problem** that barrier represent for you (*severity*)  If the barrier is not a problem for you, circle ‘never’ (i.e., ‘0’) and move to the next item. | | Never  Rarely | | Sometimes | Often | Very often | Slight | Moderate | Severe |
| 1 | Challenging behaviours exhibited by the  patient. | 0 | 1 | 2 | 3 | 4 | 1 | 2 | 3 |
| 2 | There are communication difficulties. | 0 | 1 | 2 | 3 | 4 | 1 | 2 | 3 |
| 3 | The patient’s reactivity to the healthcare  environment. | 0 | 1 | 2 | 3 | 4 | 1 | 2 | 3 |
| 4 | Lengthy waiting room time for patients on the autism spectrum. | 0 | 1 | 2 | 3 | 4 | 1 | 2 | 3 |
| 5 | The patients’ use of outside providers  (specialists, chiropractors, therapists etc.). | 0 | 1 | 2 | 3 | 4 | 1 | 2 | 3 |
| 6 | Consultations are too short to  accommodate patients on the autism spectrum. | 0 | 1 | 2 | 3 | 4 | 1 | 2 | 3 |
| 7 | There is a lack of clarity regarding GP remit/referral. | 0 | 1 | 2 | 3 | 4 | 1 | 2 | 3 |
| 8 | There are financial disincentives due to the need  for additional time with the patient. | 0 | 1 | 2 | 3 | 4 | 1 | 2 | 3 |
| 9 | The patient’s family is sceptical of  conventional medicine (e.g., vaccines). | 0 | 1 | 2 | 3 | 4 | 1 | 2 | 3 |
| 10 | These is a lack of providers willing to work with patients on the autism spectrum. | 0 | 1 | 2 | 3 | 4 | 1 | 2 | 3 |
| 11 | Family/carer involvement makes healthcare  provision for patients on the autism spectrum more complex. | 0 | 1 | 2 | 3 | 4 | 1 | 2 | 3 |
| 12 | I prefer to avoid working with patients on the autism spectrum. | 0 | 1 | 2 | 3 | 4 | 1 | 2 | 3 |
| 13 | There is a lack of support for patients and caregivers. | 0 | 1 | 2 | 3 | 4 | 1 | 2 | 3 |
| 14 | The physical environment in healthcare settings is unsuitable for patients on the autism spectrum. | 0 | 1 | 2 | 3 | 4 | 1 | 2 | 3 |
| 15 | Lack of own knowledge regarding autism | 0 | 1 | 2 | 3 | 4 | 1 | 2 | 3 |
| 16 | There is a lack of coordination between services. | 0 | 1 | 2 | 3 | 4 | 1 | 2 | 3 |
| 17 | There are shortages of medical and non-medical services for people on the autism spectrum. | 0 | 1 | 2 | 3 | 4 | 1 | 2 | 3 |

*Note*: Due to large amounts of missing data, no analyses were conducted using the severity scale; The EFA is based on frequency scale data only.
